# Tris[2-(2-thienyl­methyl­amino)­eth­yl]ammonium triiodide

**DOI:** 10.1107/S1600536810039462

**Published:** 2010-10-09

**Authors:** Muhammet Işıklan, Frank R. Fronczek, Md. Alamgir Hossain

**Affiliations:** a Department of Chemistry and Biochemistry, Jackson State University, Jackson, MS 39217, USA; b Department of Chemistry, Louisiana State University, Baton Rouge, LA 70803, USA

## Abstract

In the title compound, C_21_H_33_N_4_S_3_^3+^·3I^−^, three secondary amines are protonated, while the central amine remains unprotonated. One thio­phene is disordered with an occupancy ratio of 0.868 (6)/0.132 (6). Each protonated amine is involved in N—H⋯I hydrogen-bonding inter­actions with the iodide anions.

## Related literature

For general background to anion hosts, see: Bianchi *et al.* (1997 ▶); Kang *et al.* (2005 ▶); Hossain (2008 ▶); For related structures, see: Bazzicalupi *et al.* (2009 ▶); Hossain *et al.* (2002 ▶, 2004 ▶); Burgess *et al.* (1991 ▶); Saeed *et al.* (2010 ▶).
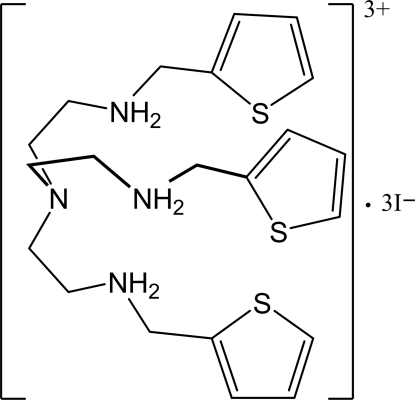


## Experimental

### 

#### Crystal data

C_21_H_33_N_4_S_3_^3+^·3I^−^
*M* *_r_* = 818.42
Orthorhombic, 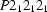

*a* = 10.5433 (5) Å
*b* = 11.4203 (6) Å
*c* = 24.5107 (15) Å
*V* = 2951.3 (3) Å^3^
*Z* = 4
Mo *K*α radiation
μ = 3.41 mm^−1^
*T* = 90 K
0.20 × 0.17 × 0.15 mm

#### Data collection

Nonius KappaCCD diffractometer with Oxford Cryostream
Absorption correction: multi-scan (*HKL *SCALEPACK**; Otwinowski & Minor, 1997 ▶) *T*_min_ = 0.549, *T*_max_ = 0.629
90447 measured reflections
10653 independent reflections
9446 reflections with *I* > 2σ(*I*)
*R*_int_ = 0.069

#### Refinement

*R*[*F* ^2^ > 2σ(*F* ^2^)] = 0.038
*wR*(*F* ^2^) = 0.093
*S* = 1.04
10653 reflections
277 parameters
30 restraints
H-atom parameters constrained
Δρ_max_ = 2.96 e Å^−3^
Δρ_min_ = −2.18 e Å^−3^
Absolute structure: Flack (1983 ▶), 4708 Friedel pairs
Flack parameter: 0.02 (2)

### 

Data collection: *COLLECT* (Nonius, 2000 ▶); cell refinement: *DENZO* and *SCALEPACK* (Otwinowski & Minor, 1997 ▶); data reduction: *DENZO* and *SCALEPACK*; program(s) used to solve structure: *SIR97* (Altomare *et al.*, 1999 ▶); program(s) used to refine structure: *SHELXL97* (Sheldrick, 2008 ▶); molecular graphics: *ORTEP–3* (Farrugia, 1997 ▶); software used to prepare material for publication: *SHELXL97*.

## Supplementary Material

Crystal structure: contains datablocks global, I. DOI: 10.1107/S1600536810039462/rk2235sup1.cif

Structure factors: contains datablocks I. DOI: 10.1107/S1600536810039462/rk2235Isup2.hkl

Additional supplementary materials:  crystallographic information; 3D view; checkCIF report

## Figures and Tables

**Table 1:** Hydrogen-bond geometry (Å, °)

*D*—H⋯*A*	*D*—H	H⋯*A*	*D*⋯*A*	*D*—H⋯*A*
N2—H21*N*⋯I1^i^	0.92	2.79	3.557 (4)	142
N2—H22*N*⋯I2	0.92	2.67	3.543 (4)	160
N3—H31*N*⋯I1	0.92	2.78	3.547 (3)	142
N3—H32*N*⋯I3	0.92	2.59	3.460 (3)	158
N4—H41*N*⋯I1	0.92	2.73	3.553 (4)	150
N4—H42*N*⋯I2^ii^	0.92	2.57	3.479 (4)	172

Symmetry codes: (i) 
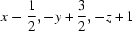
; (ii) 
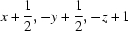
.

**Fig. 1.**
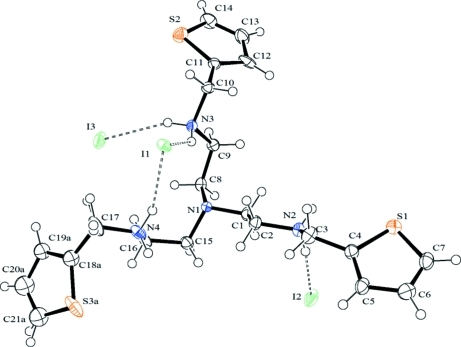
The structure of the title compound with the atom numbering scheme. Displacement ellipsoids are drawn at the 50% probability level. H atoms are presented as small spheres of arbitrary radius. Only major fragment is drawn. The several H bonds are drawn by dashed lines.

## supplementary crystallographic information

### Comment

Anions play a key role in many chemical and biological processes. In particular, structural characterization of an anion complex is important in achieving selective hosts for anions (Hossain, 2008, Saeed *et al.*, 2010). Among the numerous systems, trigonal receptors are of interest because of their synthetic simplicity and capability for anion binding though hydrogen bonding interactions. Tris(aminoethyl)–amine is an excellent building block for synthesizing fuctionalized tripodal hosts for anion binding (Burgess *et al.*, 1991; Hossain *et al.*, 2004; Bazzicalupi, *et al.*, 2009). These molecules have been shown to bind a variety of anion including nitrate, phosphate and sulfate (Bianchi *et al.*, 1997; Kang *et al.*, 2005). Herein, we report the molecular structure of the title compound in which three iodides are held by hydrogen bonding with protonated secondary amines.

Single crystal analysis of the title compound reveals that the molecule crystallizes in its orthorhombic space group forming a cavity. The tren unit is triply charged, where all three secondary N atoms are protonated. The central amine is not protonated. The three arms form a cavity, and one thiophene unit is disordered. In the complex, the protonated amines are involved in hydrogen bonding interactions with iodide anions having N···I distances 3.460 (3) to 3.553 (4)Å (Fig. 1 and Table 1). One iodide (I1) accepts two hydrogen bonds from two protonated amines (N3 and N4), while each of the other two iodides accepts one hydrogen bond from N2 and N3. Therefore, one secondary nitrogen (N3) donates two hydrogen bonds to two iodides (I1 and I3). The N···I distances are comparable with those observed in an iodide complex of an azacryptand (3.476 (4)Å and 3.632 (4)Å) reported earlier (Hossain *et al.*, 2002).

The disorder of the thiophene ring containing S3 involves two conformations, differing by rotation about two different bonds. One is a twofold rotation about C17—C18, which swaps S3 and C19. Refinement of this type of model resulted in elongated ellipsoids in the plane of the ring for all atoms of the thiophene, as well as unacceptable residual densities. This was interpreted as a second conformational difference involving a difference in rotation about the N4—C17 bond, amounting to a torsional difference of 11.7°.

### Experimental

To a solution of 2–thiophene aldehyde (4.60 g, 41 mmol) in diethylether (50 ml) was added tris(2–aminoethyl)amine (2.00 g, 13.7 mmol) in ethanol (50 ml). The mixture was stirred overnight at room temperature, and the solvent was evaporated. After diluting with methanol (100 ml), NaBH_4_ (2.00 g) was added to convert the imine into the corresponding amine. The reaction mixture was stirred for 24 hrs at room temperature. After evaporation of the solvent, the residue was partitioned in water/CH_2_Cl_2_ (50/50 ml). The organic layers were collected and dried with MgSO_4_ to give an oily product. Yield 4.38 g (74%). ^1^H NMR (500 MHz, CDCl_3_, *TMS*): *δ* 2.58 (t, 6H, NC*H*_2_), 2.69 ((t, 6H, NCH_2_C*H*_2_), 3.95 (s, 6H, *Ar*C*H*_2_)), 6.89 (b, 3H, *Ar**H*), 6.93 (b, *Ar**H*), 7.18 (b, *Ar**H*). MS (ESI): m/*z* (+) 435 (*M* + H)^+^. The iodide salt was prepared from the reaction of the free amine (0.20 g, 0.47 mmol) with HI in ethanol. The white precipitate was obtained after evaporation of the solvent. The salt was redissolved in water and ethanol (1:2 *v*/*v*, 1 ml) and crystals suitable for X–ray analysis were grown from slow evaporation of the solvent at room temperature.

### Refinement

H atoms based on C were placed in idealized positions with C—H distances 0.95Å–0.99Å, N—H distances 0.92Å, and thereafter treated as riding. *U*_iso_ for H was assigned as 1.2 times *U*_eq_ of the attached atom. The largest residual density peak was 0.81Å from I2, and the deepest hole was 0.59Å from I2. The disorder in the thiophene ring containing S3 was modeled with two orientations having populations 0.868 (6) and 0.132 (6), their geometries being restrained to be the same as that of the thiophene containing S1. This required 30 restraints. Full anisotropic refinement was not successful for the disordered region, and it was necessary to treat nine atoms as isotropic, with a common displacement parameter for the five atoms of the minor contributor thiophene ring. The absolute structure was determined by refinement of the Flack (1983) parameter, based on 4708 Friedel pairs. Six low–angle reflections were given zero weight in the refinement.

### Crystal data

**Table:**

C_21_H_33_N_4_S_3_^3+^·3I^−^	*F*(000) = 1576
*M**_r_* = 818.42	*D*_x_ = 1.842 Mg m^−^^3^
Orthorhombic, *P*2_1_2_1_2_1_	Mo *K*α radiation, λ = 0.71073 Å
Hall symbol: P 2ac 2ab	Cell parameters from 5918 reflections
*a* = 10.5433 (5) Å	θ = 2.5–32.6°
*b* = 11.4203 (6) Å	µ = 3.41 mm^−^^1^
*c* = 24.5107 (15) Å	*T* = 90 K
*V* = 2951.3 (3) Å^3^	Block, colourless
*Z* = 4	0.20 × 0.17 × 0.15 mm

### Data collection

**Table:**

Nonius KappaCCD diffractometer with Oxford Cryostream	10653 independent reflections
Radiation source: fine–focus sealed tube	9446 reflections with *I* > 2σ(*I*)
graphite	*R*_int_ = 0.069
ω– and φ–scans	θ_max_ = 32.6°, θ_min_ = 2.6°
Absorption correction: multi-scan (*HKL *SCALEPACK**; Otwinowski & Minor, 1997)	*h* = −15→15
*T*_min_ = 0.549, *T*_max_ = 0.629	*k* = −17→17
90447 measured reflections	*l* = −36→36

### Refinement

**Table:**

Refinement on *F*^2^	Secondary atom site location: difference Fourier map
Least-squares matrix: full	Hydrogen site location: inferred from neighbouring sites
*R*[*F*^2^ > 2σ(*F*^2^)] = 0.038	H-atom parameters constrained
*wR*(*F*^2^) = 0.093	*w* = 1/[σ^2^(*F*_o_^2^) + (0.0425*P*)^2^ + 8.7643*P*] where *P* = (*F*_o_^2^ + 2*F*_c_^2^)/3
*S* = 1.04	(Δ/σ)_max_ = 0.003
10653 reflections	Δρ_max_ = 2.96 e Å^−^^3^
277 parameters	Δρ_min_ = −2.18 e Å^−^^3^
30 restraints	Absolute structure: Flack (1983), 4708 Friedel pairs
Primary atom site location: structure-invariant direct methods	Flack parameter: 0.02 (2)

### Special details

**Table:**

Geometry. All s.u.'s (except the s.u. in the dihedral angle between two l.s. planes) are estimated using the full covariance matrix. The cell s.u.'s are taken into account individually in the estimation of s.u.'s in distances, angles and torsion angles; correlations between s.u.'s in cell parameters are only used when they are defined by crystal symmetry. An approximate (isotropic) treatment of cell s.u.'s is used for estimating s.u.'s involving l.s. planes.
Refinement. Refinement of *F*^2^ against ALL reflections. The weighted *R*–factor *wR* and goodness of fit *S* are based on *F*^2^, conventional *R*–factors *R* are based on *F*, with *F* set to zero for negative *F*^2^. The threshold expression of *F*^2^ > σ(*F*^2^) is used only for calculating *R*–factors(gt) *etc*. and is not relevant to the choice of reflections for refinement. *R*–factors based on *F*^2^ are statistically about twice as large as those based on *F*, and *R*–factors based on ALL data will be even larger.

### Fractional atomic coordinates and isotropic or equivalent isotropic displacement parameters (Å^2^)

**Table:**

	*x*	*y*	*z*	*U*_iso_*/*U*_eq_	Occ. (<1)
I1	0.66009 (2)	0.80566 (2)	0.512489 (10)	0.01937 (5)	
I2	0.28784 (4)	0.15756 (3)	0.526628 (15)	0.03742 (9)	
I3	0.69376 (3)	0.78787 (3)	0.708662 (14)	0.03409 (8)	
S2	0.31359 (12)	1.01963 (11)	0.68942 (5)	0.0312 (2)	
N1	0.4937 (3)	0.5219 (3)	0.59749 (14)	0.0169 (6)	
N2	0.3384 (4)	0.4343 (3)	0.46508 (14)	0.0209 (6)	
H21N	0.2630	0.4723	0.4718	0.025*	
H22N	0.3266	0.3558	0.4717	0.025*	
N3	0.4390 (3)	0.7677 (3)	0.62014 (14)	0.0179 (6)	
H31N	0.4863	0.7388	0.5916	0.021*	
H32N	0.4946	0.7922	0.6467	0.021*	
N4	0.7721 (4)	0.5480 (4)	0.57790 (16)	0.0247 (7)	
H41N	0.7136	0.6020	0.5660	0.030*	
H42N	0.7851	0.4954	0.5500	0.030*	
C1	0.3954 (4)	0.4734 (4)	0.56157 (18)	0.0210 (8)	
H1A	0.3155	0.5180	0.5662	0.025*	
H1B	0.3789	0.3907	0.5713	0.025*	
C2	0.4394 (4)	0.4811 (4)	0.50268 (17)	0.0225 (8)	
H2A	0.4580	0.5637	0.4933	0.027*	
H2B	0.5183	0.4352	0.4980	0.027*	
C3	0.3761 (5)	0.4522 (4)	0.40606 (18)	0.0273 (9)	
H3A	0.3702	0.5367	0.3973	0.033*	
H3B	0.4657	0.4283	0.4015	0.033*	
S1	0.13415 (11)	0.40416 (11)	0.36040 (5)	0.0283 (2)	
C4	0.2957 (4)	0.3848 (4)	0.36614 (18)	0.0251 (8)	
C5	0.3416 (5)	0.3115 (4)	0.32588 (17)	0.0267 (8)	
H5	0.4283	0.2907	0.3217	0.032*	
C6	0.2417 (5)	0.2710 (4)	0.29130 (19)	0.0315 (10)	
H6	0.2547	0.2187	0.2617	0.038*	
C7	0.1270 (5)	0.3147 (4)	0.30498 (19)	0.0296 (9)	
H7	0.0509	0.2974	0.2858	0.036*	
C8	0.4383 (4)	0.5616 (4)	0.64961 (17)	0.0205 (7)	
H8A	0.5074	0.5772	0.6760	0.025*	
H8B	0.3845	0.4985	0.6648	0.025*	
C9	0.3594 (4)	0.6709 (4)	0.64269 (16)	0.0196 (7)	
H9A	0.2878	0.6549	0.6177	0.024*	
H9B	0.3240	0.6948	0.6784	0.024*	
C10	0.3644 (4)	0.8723 (4)	0.60019 (17)	0.0202 (8)	
H10A	0.4240	0.9363	0.5908	0.024*	
H10B	0.3179	0.8505	0.5666	0.024*	
C11	0.2721 (4)	0.9156 (4)	0.64173 (17)	0.0209 (8)	
C12	0.1429 (5)	0.8797 (5)	0.64632 (19)	0.0309 (11)	
H12	0.1017	0.8239	0.6237	0.037*	
C13	0.0854 (5)	0.9420 (5)	0.6908 (2)	0.0340 (11)	
H13	−0.0005	0.9312	0.7013	0.041*	
C14	0.1650 (5)	1.0182 (5)	0.7170 (2)	0.0336 (10)	
H14	0.1403	1.0651	0.7472	0.040*	
C15	0.5898 (4)	0.4310 (4)	0.6089 (2)	0.0243 (8)	
H15A	0.6016	0.3819	0.5760	0.029*	
H15B	0.5590	0.3798	0.6387	0.029*	
C16	0.7168 (4)	0.4837 (4)	0.62527 (18)	0.0235 (8)	
H16A	0.7049	0.5382	0.6563	0.028*	
H16B	0.7755	0.4208	0.6370	0.028*	
C17	0.8947 (4)	0.6105 (5)	0.5889 (2)	0.0337 (11)	
H17A	0.8785	0.6789	0.6127	0.040*	0.868 (6)
H17B	0.9304	0.6396	0.5541	0.040*	0.868 (6)
H17C	0.8735	0.6876	0.6050	0.040*	0.132 (6)
H17D	0.9355	0.6258	0.5532	0.040*	0.132 (6)
S3A	1.04963 (14)	0.40627 (17)	0.58903 (9)	0.0441 (6)	0.868 (6)
C18A	0.9893 (7)	0.5305 (6)	0.6160 (3)	0.0322 (13)*	0.868 (6)
C19A	1.0385 (6)	0.5521 (6)	0.6685 (3)	0.0331 (12)*	0.868 (6)
H19A	1.0177	0.6178	0.6905	0.040*	0.868 (6)
C20A	1.1234 (8)	0.4622 (7)	0.6835 (3)	0.0493 (18)*	0.868 (6)
H20A	1.1680	0.4617	0.7172	0.059*	0.868 (6)
C21A	1.1351 (8)	0.3783 (7)	0.6464 (3)	0.0448 (17)*	0.868 (6)
H21A	1.1856	0.3103	0.6513	0.054*	0.868 (6)
S3B	1.0412 (10)	0.6016 (10)	0.6856 (4)	0.033 (3)*	0.132 (6)
C18B	0.993 (4)	0.554 (3)	0.6254 (12)	0.033 (3)*	0.132 (6)
C19B	1.054 (4)	0.447 (3)	0.6152 (12)	0.033 (3)*	0.132 (6)
H19B	1.0474	0.4043	0.5819	0.040*	0.132 (6)
C20B	1.128 (4)	0.408 (2)	0.6616 (14)	0.033 (3)*	0.132 (6)
H20B	1.1692	0.3344	0.6640	0.040*	0.132 (6)
C21B	1.132 (3)	0.489 (3)	0.7001 (13)	0.033 (3)*	0.132 (6)
H21B	1.1821	0.4833	0.7323	0.040*	0.132 (6)

### Atomic displacement parameters (Å^2^)

**Table:**

	*U*^11^	*U*^22^	*U*^33^	*U*^12^	*U*^13^	*U*^23^
I1	0.01816 (10)	0.02106 (11)	0.01888 (10)	0.00339 (9)	0.00220 (9)	0.00444 (9)
I2	0.0504 (2)	0.02224 (13)	0.03961 (17)	−0.01073 (13)	−0.01876 (15)	0.00445 (12)
I3	0.03868 (16)	0.02779 (14)	0.03580 (15)	−0.00952 (12)	−0.01948 (13)	0.00415 (12)
S2	0.0324 (6)	0.0314 (6)	0.0298 (5)	0.0004 (5)	0.0018 (5)	−0.0100 (4)
N1	0.0152 (14)	0.0155 (14)	0.0200 (15)	0.0018 (12)	−0.0020 (12)	−0.0027 (12)
N2	0.0193 (14)	0.0213 (15)	0.0221 (15)	−0.0023 (13)	−0.0029 (13)	−0.0018 (12)
N3	0.0152 (14)	0.0196 (15)	0.0189 (15)	−0.0007 (11)	−0.0005 (12)	−0.0024 (12)
N4	0.0216 (17)	0.0277 (18)	0.0248 (17)	0.0067 (14)	0.0015 (14)	0.0108 (14)
C1	0.0190 (18)	0.0216 (19)	0.0223 (18)	−0.0027 (15)	−0.0032 (15)	0.0002 (15)
C2	0.0193 (17)	0.0244 (19)	0.024 (2)	−0.0032 (15)	0.0001 (14)	−0.0034 (15)
C3	0.034 (2)	0.030 (2)	0.0179 (18)	−0.0056 (18)	0.0019 (17)	0.0004 (16)
S1	0.0268 (6)	0.0249 (5)	0.0334 (6)	0.0016 (4)	−0.0023 (4)	−0.0020 (4)
C4	0.0248 (19)	0.0242 (19)	0.026 (2)	−0.0003 (17)	−0.0041 (17)	0.0008 (16)
C5	0.033 (2)	0.026 (2)	0.0215 (17)	−0.0029 (19)	−0.0073 (16)	0.0021 (16)
C6	0.047 (3)	0.028 (2)	0.0199 (19)	0.0038 (19)	−0.0049 (19)	0.0035 (17)
C7	0.035 (2)	0.027 (2)	0.027 (2)	−0.0053 (18)	−0.0090 (17)	0.0038 (17)
C8	0.0180 (17)	0.0239 (19)	0.0196 (18)	−0.0032 (14)	−0.0006 (14)	0.0003 (15)
C9	0.0171 (17)	0.0211 (18)	0.0208 (17)	−0.0004 (13)	0.0043 (13)	−0.0005 (14)
C10	0.023 (2)	0.0185 (17)	0.0191 (17)	0.0024 (14)	0.0001 (14)	−0.0026 (13)
C11	0.0221 (19)	0.0207 (18)	0.0200 (18)	0.0051 (15)	−0.0031 (15)	−0.0042 (14)
C12	0.030 (2)	0.040 (3)	0.0221 (19)	0.024 (2)	0.0050 (17)	−0.0012 (18)
C13	0.025 (2)	0.044 (3)	0.034 (2)	0.010 (2)	−0.0009 (19)	−0.008 (2)
C14	0.032 (2)	0.042 (3)	0.027 (2)	0.012 (2)	0.0066 (19)	−0.0079 (19)
C15	0.0193 (19)	0.023 (2)	0.030 (2)	0.0009 (15)	0.0007 (15)	0.0011 (17)
C16	0.0158 (18)	0.027 (2)	0.027 (2)	0.0045 (15)	0.0024 (15)	0.0129 (16)
C17	0.0174 (19)	0.043 (3)	0.041 (3)	0.0009 (18)	−0.0022 (19)	0.024 (2)
S3A	0.0175 (6)	0.0484 (11)	0.0664 (13)	0.0046 (6)	0.0020 (7)	0.0329 (9)

### Geometric parameters (Å, °)

**Table:**

S2—C14	1.706 (5)	C9—H9B	0.9900
S2—C11	1.723 (4)	C10—C11	1.493 (6)
N1—C1	1.468 (5)	C10—H10A	0.9900
N1—C8	1.476 (5)	C10—H10B	0.9900
N1—C15	1.477 (6)	C11—C12	1.427 (7)
N2—C2	1.506 (5)	C12—C13	1.436 (7)
N2—C3	1.514 (6)	C12—H12	0.9500
N2—H21N	0.9200	C13—C14	1.368 (8)
N2—H22N	0.9200	C13—H13	0.9500
N3—C9	1.493 (5)	C14—H14	0.9500
N3—C10	1.512 (5)	C15—C16	1.522 (6)
N3—H31N	0.9200	C15—H15A	0.9900
N3—H32N	0.9200	C15—H15B	0.9900
N4—C16	1.492 (5)	C16—H16A	0.9900
N4—C17	1.501 (6)	C16—H16B	0.9900
N4—H41N	0.9200	C17—C18A	1.507 (8)
N4—H42N	0.9200	C17—C18B	1.51 (2)
C1—C2	1.519 (6)	C17—H17A	0.9900
C1—H1A	0.9900	C17—H17B	0.9900
C1—H1B	0.9900	C17—H17C	0.9900
C2—H2A	0.9900	C17—H17D	0.9900
C2—H2B	0.9900	S3A—C18A	1.690 (6)
C3—C4	1.506 (6)	S3A—C21A	1.700 (7)
C3—H3A	0.9900	C18A—C19A	1.409 (8)
C3—H3B	0.9900	C19A—C20A	1.411 (9)
S1—C7	1.702 (5)	C19A—H19A	0.9500
S1—C4	1.724 (5)	C20A—C21A	1.327 (10)
C4—C5	1.382 (6)	C20A—H20A	0.9500
C5—C6	1.429 (6)	C21A—H21A	0.9500
C5—H5	0.9500	S3B—C21B	1.641 (16)
C6—C7	1.350 (7)	S3B—C18B	1.655 (16)
C6—H6	0.9500	C18B—C19B	1.406 (17)
C7—H7	0.9500	C19B—C20B	1.448 (17)
C8—C9	1.510 (6)	C19B—H19B	0.9500
C8—H8A	0.9900	C20B—C21B	1.322 (17)
C8—H8B	0.9900	C20B—H20B	0.9500
C9—H9A	0.9900	C21B—H21B	0.9500
			
C14—S2—C11	91.7 (2)	H10A—C10—H10B	107.9
C1—N1—C8	110.8 (3)	C12—C11—C10	125.5 (4)
C1—N1—C15	109.5 (3)	C12—C11—S2	112.8 (3)
C8—N1—C15	108.9 (3)	C10—C11—S2	121.7 (3)
C2—N2—C3	110.6 (3)	C11—C12—C13	108.7 (5)
C2—N2—H21N	109.5	C11—C12—H12	125.7
C3—N2—H21N	109.5	C13—C12—H12	125.7
C2—N2—H22N	109.5	C14—C13—C12	114.3 (5)
C3—N2—H22N	109.5	C14—C13—H13	122.8
H21N—N2—H22N	108.1	C12—C13—H13	122.8
C9—N3—C10	114.3 (3)	C13—C14—S2	112.6 (4)
C9—N3—H31N	108.7	C13—C14—H14	123.7
C10—N3—H31N	108.7	S2—C14—H14	123.7
C9—N3—H32N	108.7	N1—C15—C16	112.1 (4)
C10—N3—H32N	108.7	N1—C15—H15A	109.2
H31N—N3—H32N	107.6	C16—C15—H15A	109.2
C16—N4—C17	115.5 (4)	N1—C15—H15B	109.2
C16—N4—H41N	108.4	C16—C15—H15B	109.2
C17—N4—H41N	108.4	H15A—C15—H15B	107.9
C16—N4—H42N	108.4	N4—C16—C15	109.5 (4)
C17—N4—H42N	108.4	N4—C16—H16A	109.8
H41N—N4—H42N	107.5	C15—C16—H16A	109.8
N1—C1—C2	109.4 (3)	N4—C16—H16B	109.8
N1—C1—H1A	109.8	C15—C16—H16B	109.8
C2—C1—H1A	109.8	H16A—C16—H16B	108.2
N1—C1—H1B	109.8	N4—C17—C18A	111.1 (5)
C2—C1—H1B	109.8	N4—C17—C18B	119.4 (19)
H1A—C1—H1B	108.2	N4—C17—H17A	109.4
N2—C2—C1	110.2 (3)	C18A—C17—H17A	109.4
N2—C2—H2A	109.6	C18B—C17—H17A	96.2
C1—C2—H2A	109.6	N4—C17—H17B	109.4
N2—C2—H2B	109.6	C18A—C17—H17B	109.4
C1—C2—H2B	109.6	C18B—C17—H17B	113.2
H2A—C2—H2B	108.1	H17A—C17—H17B	108.0
C4—C3—N2	113.8 (4)	N4—C17—H17C	107.5
C4—C3—H3A	108.8	C18A—C17—H17C	120.9
N2—C3—H3A	108.8	C18B—C17—H17C	107.5
C4—C3—H3B	108.8	H17B—C17—H17C	97.5
N2—C3—H3B	108.8	N4—C17—H17D	107.5
H3A—C3—H3B	107.7	C18A—C17—H17D	102.1
C7—S1—C4	91.8 (2)	C18B—C17—H17D	107.5
C5—C4—C3	125.2 (4)	H17A—C17—H17D	117.2
C5—C4—S1	111.5 (3)	H17C—C17—H17D	107.0
C3—C4—S1	122.9 (4)	C18A—S3A—C21A	91.9 (3)
C4—C5—C6	111.2 (4)	C19A—C18A—C17	122.7 (6)
C4—C5—H5	124.4	C19A—C18A—S3A	111.4 (5)
C6—C5—H5	124.4	C17—C18A—S3A	125.8 (5)
C7—C6—C5	113.2 (4)	C18A—C19A—C20A	110.2 (6)
C7—C6—H6	123.4	C18A—C19A—H19A	124.9
C5—C6—H6	123.4	C20A—C19A—H19A	124.9
C6—C7—S1	112.3 (4)	C21A—C20A—C19A	113.9 (7)
C6—C7—H7	123.8	C21A—C20A—H20A	123.0
S1—C7—H7	123.8	C19A—C20A—H20A	123.0
N1—C8—C9	112.0 (3)	C20A—C21A—S3A	112.5 (6)
N1—C8—H8A	109.2	C20A—C21A—H21A	123.8
C9—C8—H8A	109.2	S3A—C21A—H21A	123.8
N1—C8—H8B	109.2	C21B—S3B—C18B	96.6 (11)
C9—C8—H8B	109.2	C19B—C18B—C17	125.6 (18)
H8A—C8—H8B	107.9	C19B—C18B—S3B	107.7 (14)
N3—C9—C8	110.1 (3)	C17—C18B—S3B	126.6 (17)
N3—C9—H9A	109.6	C18B—C19B—C20B	111.8 (16)
C8—C9—H9A	109.6	C18B—C19B—H19B	124.1
N3—C9—H9B	109.6	C20B—C19B—H19B	124.1
C8—C9—H9B	109.6	C21B—C20B—C19B	111.5 (17)
H9A—C9—H9B	108.1	C21B—C20B—H20B	124.2
C11—C10—N3	112.3 (3)	C19B—C20B—H20B	124.2
C11—C10—H10A	109.1	C20B—C21B—S3B	111.9 (15)
N3—C10—H10A	109.1	C20B—C21B—H21B	124.0
C11—C10—H10B	109.1	S3B—C21B—H21B	124.0
N3—C10—H10B	109.1		
			
C8—N1—C1—C2	156.4 (4)	C1—N1—C15—C16	156.8 (4)
C15—N1—C1—C2	−83.5 (4)	C8—N1—C15—C16	−82.0 (4)
C3—N2—C2—C1	174.6 (4)	C17—N4—C16—C15	176.8 (4)
N1—C1—C2—N2	−178.7 (3)	N1—C15—C16—N4	−66.4 (5)
C2—N2—C3—C4	167.0 (4)	C16—N4—C17—C18A	50.2 (6)
N2—C3—C4—C5	−128.0 (5)	C16—N4—C17—C18B	38.5 (15)
N2—C3—C4—S1	60.0 (5)	N4—C17—C18A—C19A	−118.1 (7)
C7—S1—C4—C5	0.3 (4)	C18B—C17—C18A—C19A	12 (9)
C7—S1—C4—C3	173.3 (4)	N4—C17—C18A—S3A	61.9 (7)
C3—C4—C5—C6	−173.7 (4)	C18B—C17—C18A—S3A	−168 (10)
S1—C4—C5—C6	−0.9 (5)	C21A—S3A—C18A—C19A	2.0 (6)
C4—C5—C6—C7	1.1 (6)	C21A—S3A—C18A—C17	−178.0 (7)
C5—C6—C7—S1	−0.9 (6)	C17—C18A—C19A—C20A	179.3 (7)
C4—S1—C7—C6	0.3 (4)	S3A—C18A—C19A—C20A	−0.7 (8)
C1—N1—C8—C9	−71.3 (4)	C18A—C19A—C20A—C21A	−1.4 (10)
C15—N1—C8—C9	168.2 (3)	C19A—C20A—C21A—S3A	3.0 (10)
C10—N3—C9—C8	167.4 (3)	C18A—S3A—C21A—C20A	−2.9 (7)
N1—C8—C9—N3	−59.3 (4)	N4—C17—C18B—C19B	62 (5)
C9—N3—C10—C11	51.0 (4)	C18A—C17—C18B—C19B	7(6)
N3—C10—C11—C12	−91.8 (5)	N4—C17—C18B—S3B	−114 (3)
N3—C10—C11—S2	89.5 (4)	C18A—C17—C18B—S3B	−169 (13)
C14—S2—C11—C12	0.6 (4)	C21B—S3B—C18B—C19B	−2(4)
C14—S2—C11—C10	179.4 (4)	C21B—S3B—C18B—C17	175 (4)
C10—C11—C12—C13	−179.4 (4)	C17—C18B—C19B—C20B	−172 (4)
S2—C11—C12—C13	−0.6 (5)	S3B—C18B—C19B—C20B	5(5)
C11—C12—C13—C14	0.3 (6)	C18B—C19B—C20B—C21B	−7(6)
C12—C13—C14—S2	0.1 (6)	C19B—C20B—C21B—S3B	5(5)
C11—S2—C14—C13	−0.4 (4)	C18B—S3B—C21B—C20B	−2(4)

### Hydrogen-bond geometry (Å, °)

**Table:**

*D*—H···*A*	*D*—H	H···*A*	*D*···*A*	*D*—H···*A*
N2—H21N···I1^i^	0.92	2.79	3.557 (4)	142
N2—H22N···I2	0.92	2.67	3.543 (4)	160
N3—H31N···I1	0.92	2.78	3.547 (3)	142
N3—H32N···I3	0.92	2.59	3.460 (3)	158
N4—H41N···I1	0.92	2.73	3.553 (4)	150
N4—H42N···I2^ii^	0.92	2.57	3.479 (4)	172

Symmetry codes: (i) *x*−1/2, −*y*+3/2, −*z*+1; (ii) *x*+1/2, −*y*+1/2, −*z*+1.
